# A Dictionary of Virology, Third Edition 

**DOI:** 10.3201/eid0806.020058

**Published:** 2002-06

**Authors:** Charles H. Calisher

**Affiliations:** Colorado State University, Fort Collins, Colorado

A dictionary is a reference book containing an alphabetical list of words, with information about each word; it usually includes meaning, pronunciation, and etymology. The third edition of Brian Mahy’s Dictionary of Virology provides definitions of words commonly and uncommonly used in the field of virology, words that so often divide us into subgroups. Given that new words are associated with new thought processes and the new procedures that accompany them, picking up the latest technical terms and buzzwords is difficult. In addition, these words are added to the astounding number of words we have all accumulated over 10, 20, 30, or 40 years, so that the sum can be overwhelming. Thus, having a book containing all these words is functional and valuable. One can run to a secluded place, check this book, and thereafter appear to know what a graduate student is talking about. Previous editions were available in the United States in hardcover only, but this latest edition is in a convenient paperback form.

This book is not a complete dictionary in the classical sense; it does not provide a pronunciation guide as a dictionary might. Mahy prefaces the book with the disclaimer: “Viruses which only infect bacteria, fungi, invertebrates or plants are outside the scope of this Dictionary.” Perhaps the publisher drew the line at 422 pages, or perhaps this book should have been named “Dictionary of (for the most part) Animal Viruses”. In any event, if you are a molecular biologist, clinician, or epidemiologist involved in work with animal viruses, a graduate student, a senior faculty member who is overly specialized, a journalist, or a layman without a life, this book is highly recommended. 

A Dictionary of Virology includes useful, informative, and concise definitions and brief descriptions of a multitude of words and terms we use each day, and it also contains words used by others. The book’s entries include not only the necessary dry descriptions but also miniexplanations of very complex matters. Key references will lead you in the right direction, should you want to know more.

As might be expected of a book containing this much information, there are some minor shortcomings. For example, names of virus strains are presented for some viruses without explanation as to why names of virus strains are not presented for all viruses. Virus (= species) names are promised as conforming to the latest taxonomic vogue (i.e., italics), but not all species names are italicized. The definition for *Highlands J virus* indicates that this is not the etiologic agent of disease (disease in horses and turkeys illustrates otherwise). The dictionary alleges that lyssa virus is to be a synonym for *Rabies virus*, but whereas *Rabies virus* is a lyssavirus (a member of the genus *Lyssavirus*), there are many lyssaviruses, only one of which is *Rabies virus*. *Trivittatus virus* is defined as a member of the “California encephalitis virus serogroup,” but at least this week, it is considered a serotype of the species *California encephalitis virus* (California serogroup). A few spelling errors occur, which, I am certain, will be corrected in the fourth edition.

Never mind the minor and few errors of commission, there are few, if any, (I looked) gross errors of omission. A Dictionary of Virology, therefore, is not only unique, it is useful**—**quite remarkable for a dictionary—pleasant and informative reading. Do not keep this book on your nightstand; by the time you get to it, it will be too late in the day. Instead, carry it in your attaché case or in the glove compartment of your pickup truck. This book reflects the remarkable diversity of viruses and of the terms that have been coined to discuss them so that we may have the requisite common language. I recommend it highly.

